# Mastering Pediatric Airway Skills: A Procedural Simulation Using Rapid Cycle Deliberate Practice for Emergency Medicine Residents

**DOI:** 10.1002/aet2.70180

**Published:** 2026-05-11

**Authors:** Kei U. Wong, Isabel T. Gross

**Affiliations:** ^1^ Department of Emergency Medicine, Division of Pediatric Emergency Medicine Rutgers New Jersey Medical School Newark New Jersey USA; ^2^ Department of Pediatrics, Section of Emergency Medicine Yale University School of Medicine New Haven Connecticut USA

## Abstract

**Background:**

Pediatric emergency medicine (PEM) training within emergency medicine (EM) residency programs varies widely in preceptor expertise, critical care exposure, and procedural opportunities. Despite the importance of pediatric airway management, real‐world exposure to critically ill children remains limited and inconsistent across training sites. Current ACGME guidelines provide minimal direction on pediatric‐specific content or mandatory procedures, leaving gaps in emergent airway training.

**Objective of the Innovation:**

To address perceived deficiencies in pediatric airway skills among EM residents, we implemented a longitudinal, simulation‐based pediatric airway curriculum. The primary goal was to improve pediatric airway knowledge, confidence, procedural competency in airway equipment setup, bag‐valve‐mask (BVM) ventilation, troubleshooting, intubation techniques using a flipped classroom model, and rapid cycle deliberate practice (RCDP).

**Development Process and Implementation:**

Guided by Kern's curriculum design framework and selected components of Sawyer's six‐step procedural learning model, the curriculum incorporated asynchronous learning and eight interactive simulation sessions over a 12‐month period. 40 residents in four postgraduate years participated in two individualized RCDP sessions annually, supported by faculty trained in pediatric airway management. A novel checklist was used for objective performance assessment.

**Outcomes:**

Thirty‐one residents completed baseline and post‐intervention procedural assessments. Significant improvements were observed in equipment setup and intubation steps (*p* < 0.05), with first‐pass intubation time reduced from 9 min and 12 s to 7 min and 9 s (*p* < 0.05). Self‐reported confidence in pediatric airway management increased across all PGY levels (*p* < 0.05), and knowledge scores improved (7.8 vs. 9.7, *p* < 0.05). Resident feedback strongly endorsed the curriculum's value in building skills and confidence.

**Conclusion:**

A structured, simulation‐based pediatric airway curriculum using RCDP effectively enhances EM residents' procedural performance and confidence. This model offers a scalable approach for addressing critical skill gaps in PEM training. Future directions include integrating advanced airway techniques and longitudinal retention assessments.

## 1 Need for Innovation

Pediatric training is an essential component of emergency medicine (EM) residency. While the Accreditation Council for Graduate Medical Education (ACGME) proposed the requirement of 24 weeks of pediatric exposure, with at least 12 weeks in the pediatric emergency department, education and clinical exposure related to pediatric topics vary across residency programs [1].

Developing competency in a multitude of high‐acuity low‐occurrence (HALO) resuscitation procedures, including emergent pediatric airway intubation, is an integral component of EM training and practice. Yet, previous data suggest that the need for pediatric endotracheal intubation arises in the ED from 0.6 to 3.3 cases per thousand [2, 3], making clinical opportunities for EM physicians to manage pediatric airways extremely limited. The heterogeneity in the acuity spectra of pediatric illness poses a significant challenge to EM training programs. In one recent study, EM program directors reported less confidence in their graduating residents' competence in caring for pediatric patients compared with adult patients [4].

The attainment of procedural competency to proficiency requires continued focus and practice. A limited number of curricula on pediatric topics for general emergency medicine residents have been described in the literature, and more are needed to accommodate the diverse characteristics of EM resident learners. Such need only furthers the demand for procedural simulation as a key strategy in skill acquisition. Further incorporation of procedural‐focused simulation into residency curriculum has been shown to improve trainee confidence and objective performance outcomes [5, 6]. Dedicated, scheduled simulation is a cornerstone of EM residency training, where pediatric emergency medicine content should be integrated. Events that are often referred to as high‐acuity low‐occurrence, including pediatric intubation, have shown improved performance and mastery after a simulated curriculum [7, 8].

While repetition of procedural skills can help to reinforce techniques, simply performing airway procedures in the clinical setting or a one‐time simulation workshop is insufficient to attain proficiency [9]. To address pediatric gaps in EM training, we implemented a pilot longitudinal simulation curriculum to improve learners' knowledge and skills in pediatric airway management using multiple learning modalities. These modalities include a flipped classroom format with review materials and hands‐on practice with mentored procedures using rapid cycle deliberate practice (RCDP) with direct coaching to provide micro‐debriefs on airway preparedness and intubation skill sets.

In contrast to traditional debriefing, which focuses on learning after completion of a simulated scenario, RCDP is a simulation‐based curriculum that presents participants with rounds of increasing difficulty in rapid repetition, interspersing brief, direct feedback within the simulation. RCDP has demonstrated to be an effective method for teaching procedural skills [10]. One study compared RCDP to post‐simulation feedback in teaching the skill of pediatric intubations to medical students [11]. Gross et al. found overall significant improvement in scores for intubation choreography on a simulation checklist in the RCDP group. Integrating RCDP into pediatric airway education is a novel approach that could strengthen resident pediatric airway management performance.

## 2 Background

Pediatric emergency medicine (PEM) training in emergency medicine residency programs is diverse in preceptors, critical care exposure, and procedural training [12]. Despite the importance of adequate training in PEM, the exposure to critically ill or injured children requiring emergent management is often limited and sporadic across training sites. In the United States, the Society of American Emergency Physician had established a Pediatric Education Training Task Force in 1995, seeking to manage the educational experiences of EM residents in the field of pediatric EM [13]. Despite the recent proposed changes to EM residency program requirements, the ACGME provides limited guidance on specific pediatric content or mandatory procedures, including emergent airway management, that should be taught during EM residency training [1].

In the United States, over 85% of the 36 million children access emergency care annually through the general emergency departments [14]. While there is a growing number of specialized PEM physicians, the majority of pediatric patients are evaluated and managed by EM resident graduates. It is, therefore, imperative that EM residents be thoroughly trained to not only care for their pediatric patients, but especially in critical procedures such as emergent airway management. To date, various training models have been utilized to improve pediatric intubation skills acquisition [15]. Yet, the optimal method for teaching residents to effectively manage pediatric airway procedures, both simple and complex, is still unknown.

Pediatric airway emergencies are high‐stakes, low‐frequency events that require rapid, precise intervention. Aside from the EM Model of Clinical Practice, there is no up‐to‐date standard PEM education curriculum for EM residents, leaving residents underprepared for these emergent airway scenarios [12]. This training gap highlights the urgent need for a structured, competency‐based curriculum that incorporates simulation models and task trainers to enhance procedural proficiency. Rapid cycle Deliberate Practice (RCDP) has been identified as an effective simulation‐based educational tool, particularly for the acquisition and reinforcement of critical skills [10]. It involves learners repetitiously performing a simulation or procedure with micro‐debriefs and feedback. This allows for continuous improvement upon each iterative cycle in a dynamic learning process. Reinforcing the learner's clinical procedural experience with simulation training augments their exposure to infrequent procedures, increases success rates, improves performance, and enhances skills retention [16, 17].

## 3 Objective of Innovation

Following a needs assessment with graduating emergency medicine (EM) residents, a perceived deficiency in pediatric critical care skills, particularly emergent airway intubation, was identified. The primary objective of this educational initiative was to establish a pilot, longitudinal, simulation‐based pediatric airway curriculum and to address pediatric airway procedural competency, with a focus on pediatric airway preparedness. We sought to assess the residents' performance in appropriate pediatric airway equipment setup, bag‐valve‐mask (BVM) ventilation with adjuncts, troubleshooting, and intubation techniques. This initiative utilized multiple learning modalities, including a flipped classroom format in conjunction with the Rapid Cycle Deliberate Practice (RCDP) approach within our EM resident cohort. A novel, dedicated RCDP procedural skills checklist was used to objectively assess the learners' performance.

## 4 Development Process

Our program targeted 40 residents across a 4‐year EM training program. A needs assessment, conducted through anonymous surveys sent to graduated EM residents and departmental program leadership, identified perceived deficiencies in pediatric critical skills. Pediatric airway management, including emergent intubation and cricothyrotomy, was prioritized. Incorporating pediatric airway simulation training was intended to complement the existing EM residency curriculum, allowing for mastery of critical skills and knowledge.

To address variability in PEM experience, we developed an airway‐focused simulation curriculum grounded in Kern et al.'s approach to curriculum design and informed by selected components of Sawyer et al.'s six‐step procedural learning framework [18, 19]. Because this educational intervention was conducted exclusively in a simulated environment and did not involve clinical care with real patients, the full six‐step framework was not implemented. Instead, the curriculum emphasized components most applicable to simulation‐based learning, including cognitive preparation (“learn”), expert demonstration (“see”), and structured, coached practice (“practice”). Drawing on these frameworks, we sought to enhance learners' procedural performance in managing emergent pediatric airways through guided feedback. This allowed learners to progress through each step and exposed them to novel airway educational interventions, such as a flipped classroom format [20] and the use of RCDP with a novel, dedicated procedural checklist developed from the existing literature.

We proposed a longitudinal postgraduate year (PGY)‐directed simulation curriculum that reinforced learned skills through repeated practice. The goal for year‐directed sessions is to focus on procedural training tailored to the expected needs of each PGY class to achieve competency (i.e., more time on basic procedural review for interns vs. complex concepts for senior residents). As a result, smaller learner groups received maximal individualized coaching and opportunities for skill practice. This curriculum provided learners with multiple opportunities to apply their understanding of key concepts through both deliberate practice and simulation‐based coaching. Educators can correct learners' procedural techniques in real time. With improved proficiency, residents were expected to provide more consistent and effective emergency care for pediatric patients.

## 5 The Implementation Phase

The pediatric airway simulation curriculum was implemented over a 12‐month period within the four‐year emergency medicine (EM) residency program, which was funded by the SAEMF/Simulation Academy Novice Research Grant. From July 2024 to June 2025, the program delivered 8 interactive sessions. Each PGY class participated in two one‐on‐one procedural simulation training sessions with EM/PEM faculty, ensuring skill proficiency through spaced repetition. The faculty teaching the curriculum had adequate training and experience in pediatric emergency airway management. Residents were provided with asynchronous learning content, including pediatric airway anatomy, equipment setup and selection, and procedural videos for intubation. Residency leadership buy‐in and departmental support were critical to securing dedicated time and resources for the curriculum implementation.

Sessions were integrated into “Class Days,” where specific PGY classes were protected from clinical responsibilities for an academic half‐day after the residency educational conference. Each session focused on pediatric airway preparedness, included age‐appropriate airway equipment setup and selection, mastery of BVM and airway adjuncts, troubleshooting during airway management, and infant intubation techniques. Using the RDCP framework, facilitators provided immediate feedback and repeated practice opportunities.

Knowledge and self‐reported confidence assessments were administered immediately before and after each simulation session. The knowledge assessment evaluated key cognitive components of pediatric airway management, while confidence was measured using a Likert‐type scale assessing perceived readiness to perform pediatric airway procedures. These assessments were used to evaluate short‐term educational outcomes associated with curriculum participation.

Each group of learners was limited to their PGY class during each session. As such, the minimum number of instructors for a small group of learners was achieved. The goal for PGY‐directed sessions was to provide tailored training based on the needs or expected PGY competency level. This allowed small learner groups to receive the maximal individual deliberate practice, direct procedural feedback, and coaching of the psychomotor skills covered during these sessions. The RCDP procedural checklist served as a cognitive aid to standardize teaching scripts and ensure that key procedural techniques were covered. Preceptors provided immediate, direct observational feedback through deliberate practice and coaching as learners practiced on different‐sized airway task trainers. These simulations allowed residents to repeatedly practice techniques, enhancing muscle memory. This also enabled the preceptors to identify areas of weakness, improve learners' procedural accuracy and psychomotor skills, and mitigate improper techniques in real time. Participation in these sessions demonstrated strong resident engagement and commitment to mastering pediatric airway management skills.

## 6 Outcomes

**FIGURE 1:**
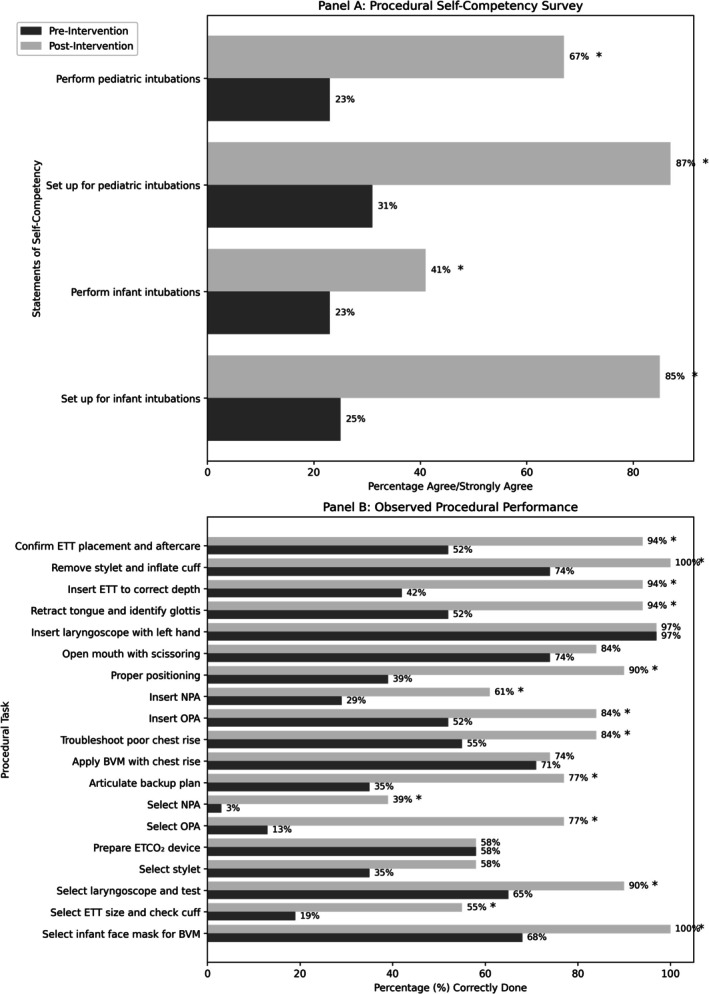
Pre‐ and post‐intervention comparison of self‐competency and procedural performance survey results. Panel A shows the percentage of participants who agreed or strongly agreed with statements of self‐competency. Panel B shows the percentage of participants correctly performing each procedural task before and after the intervention. Dark gray bars represent pre‐intervention data; light gray bars represent post‐intervention data. Asterisks (*) indicate statistically significant improvements (*p* < 0.05).

Our primary outcome was residents' pre‐and post‐intervention procedural performance, scored based on the number of items performed correctly in the RCDP procedural checklist in 3 domains: (1) age‐appropriate airway equipment selection and setup, (2) mastery of BVM and effective use of airway adjuncts, and the ability to troubleshoot during airway management, and (3) correct infant intubation techniques. The time to successful first‐pass intubation and completion of all three checklist domains was also collected. Thirty‐one residents completed baseline procedural assessments and participated in all RCDP procedural simulation training sessions (Figure 1). After curriculum implementation, all residents demonstrated a statistically significant improvement in completing checklist items related to equipment setup and intubation techniques (*p* < 0.05). Overall, residents demonstrated significant improvement across all domains, including task completion and time to successful first‐pass intubation (baseline time: 9 min and 12 s; post‐intervention time: 7 min and 9 s; *p* < 0.05).

Secondary outcomes included learners' perceived procedural confidence and knowledge in emergent pediatric airway management before and after implementation of an airway‐focused simulation curriculum. Pre‐ and post‐intervention self‐reported procedural competency surveys were completed by 39 residents. We found self‐reported confidence in pediatric airway management increased across all PGY levels (Figure 1). Analysis showed a statistically significant increase in residents' self‐perceived airway procedural competency across all four class years (*p* < 0.05) (Table S1). Knowledge assessments demonstrated improvement in understanding of pediatric airway principles and techniques among all residents (Pretest average score: 7.8; Posttest average score: 9.7; *p* < 0.05).

Feedback from residents was overwhelmingly positive, consistently affirming the curriculum's value and its role in promoting ongoing skill development (Table S2). Learners reported that simulation‐based mastery learning significantly enhanced confidence and preparedness for rare, high‐stakes pediatric airway scenarios. One resident noted, “Training through simulation definitely helps in being more comfortable,” while another shared, “Airway sim was very helpful in my pediatric airway case.” Participants emphasized the importance of repeated practice and immediate feedback, stating, “Repetition over time was helpful,” and “The RCDP and immediate feedback is practical and high yield for me.” The curriculum was also credited with improving technical proficiency and reducing cognitive load, as reflected in comments such as, “This helps me go over how to appropriately prepare for a potential pediatric intubation.” Residents highlighted that these sessions were particularly beneficial given limited clinical exposure, with one remarking, “Have not had a pediatric code thus far,” reinforcing the need for structured, simulation‐based training to bridge experience gaps. Overall, residents requested additional sessions to maintain competency and expressed interest in ongoing one‐on‐one training opportunities for other pediatric HALO procedures to sustain and enhance procedural skills. Residents reported increased pediatric airway preparation and ability to apply skills in real clinical settings. Future directions included focusing on a different pediatric critical procedure, such as cricothyrotomy, while continuing annual airway management refreshers.

## 7 Reflective Discussion

The success and potential for replication of this airway procedural training program were notable. This innovation highlighted the value of a structured, simulation‐based curriculum in addressing a critical gap in EM training. Rooted in self‐determination theory [21], the curriculum aimed to enhance EM residents' motivation through autonomy and preparation in their pediatric airway readiness. The longitudinal design of the curriculum fostered procedural proficiency and continual competency. This may have motivated learners to reconsider their clinical practice setting, such as after learning about changes to airway equipment, and to adopt best practices to improve their preparedness.

The curriculum's design, specifically its PGY‐directed format, combined with pre‐session content and the RCDP approach, made it highly adaptable for other institutions seeking to standardize pediatric airway procedural training. By targeting specific PGY levels, the curriculum can be easily scaled to accommodate larger resident cohorts without redesigning the core content while maintaining a high faculty‐to‐learner ratio during RCDP sessions for maximum engagement. The goal for year‐directed sessions was to target procedural training based on the expected needs of each PGY class to achieve competency.

The use of pre‐session materials allowed learners to progress at their own pace, maximizing limited in‐person faculty time by dedicating it to high‐value, hands‐on RCDP training. The RCDP checklist served as a reproducible teaching script that emphasized immediate correction and high repetition until procedural mastery was achieved.

Lastly, the acquisition of pediatric‐specific airway task trainers, supported by departmental funding and the SAEMF Simulation Academy Novice Grant, improved training realism and further supported resident procedural skill development.

## 8 Limitations

Given the national data indicating a widespread need for enhanced resident airway skill development in Emergency Medicine (EM), our curriculum can serve as a model for institutions seeking to bridge similar skill gaps. However, we encountered several innovation‐specific challenges and broader design limitations.

We initially experienced challenges related to resource availability and simulation realism. Specifically, we lacked an adequate number of high‐quality airway task trainers in infant and pediatric sizes. This limited the maximal individual deliberate practice, direct procedural feedback, and coaching of psychomotor skills for smaller groups of learners. Additionally, limited Pediatric Emergency Medicine (PEM) and Pediatric faculty availability constrained our session frequency, thereby restricting the number of learners by their PGY class during each training block. Time constraints during academic afternoons also required necessary curriculum adjustments. Long‐term sustainability of the curriculum depended on continued departmental and residency program leadership support.

In addition to these implementation challenges, the study's generalizability was limited by its single‐center design and small cohort of learners. The observational nature of the study precluded randomization, and the sample size was constrained by the number of learners available in the program. Outcomes were limited to short‐term measures of knowledge and self‐reported confidence; longitudinal follow‐up to assess skill retention or transfer to clinical practice was not performed. Additionally, the curriculum was not directly compared with other instructional modalities. These factors may have influenced the interpretation and applicability of findings, underscoring the need for future research with larger cohorts and randomization, incorporating longitudinal outcomes and comparative educational designs.

## 9 Conclusions

This curriculum served as an effective pediatric airway education tool for EM residents and functioned as a model for other pediatric high‐acuity, low‐occurrence (HALO) procedural training. Real‐time feedback and repeated practice were critical for skill acquisition. Future steps included incorporating advanced airway techniques (e.g., emergent cricothyroidotomy) in future iterations and exploring the integration of longitudinal assessments to evaluate skill retention.

## Author Contributions

**Kei U. Wong:** conceptualization, investigation, writing – original draft, writing – review and editing, data curation, funding acquisition, formal analysis, resources. **Isabel T. Gross:** conceptualization, writing – review and editing, supervision.

## Funding

This work was supported by the SAEMF/Simulation Academy Novice Research Grant awarded to K.U.W. at Rutgers New Jersey Medical School for simulator equipment and educational activities. Additional support was provided by the Society for Academic Emergency Medicine (AG2020‐0000000169).

## Conflicts of Interest

The authors declare no conflicts of interest.

## Supporting information


**Table S1:** Impact of RCDP simulation‐based educational intervention on procedural self‐competency and measured performance in pediatric airway management.
**Table S2:** Thematic analysis of learner feedback on confidence with airway skills.

## Data Availability

The data that supports the findings of this study are available in the Supporting Information of this article.

## References

[1] ACGME Program Requirements for Graduate Medical Education in Emergency Medicine , “Accreditation Council for Graduate Medical Education,” (2025), accessed October 5, 2025, https://www.acgme.org/globalassets/pfassets/reviewandcomment/2025/110_emergencymedicine_rc_02122025.pdf.

[2] L. D. Nguyen and S. Craig , “Paediatric Critical Procedures in the Emergency Department: Incidence, Trends and the Physician Experience,” Emergency Medicine Australasia 28, no. 1 (2016): 78–83. 26644368 10.1111/1742-6723.12514

[3] J. D. Losek , L. R. Olson , J. V. Dobson , and P. W. Glaeser , “Tracheal Intubation Practice and Maintaining Skill Competency: Survey of Pediatric Emergency Department Medical Directors,” Pediatric Emergency Care 24, no. 5 (2008): 294–299. 18496112 10.1097/PEC.0b013e31816ecbd4

[4] J. Nickerson , A. Ghatak‐Roy , K. A. Donnelly , et al., “The Current State of Pediatric Emergency Medicine Training in Emergency Medicine Residencies,” Pediatric Emergency Care 39, no. 3 (2023): 167–172. 36018727 10.1097/PEC.0000000000002819

[5] W. C. McGaghie , T. J. Draycott , W. F. Dunn , et al., “Evaluating the Impact of Simulation on Translational Patient Outcomes,” Simulation in Healthcare 6, no. Suppl (2011): S42–S47. 21705966 10.1097/SIH.0b013e318222fde9 PMC3153601

[6] E. E. Wang , J. Quinones , M. T. Fitch , et al., “Developing Technical Expertise in Emergency Medicine—The Role of Simulation in Procedural Skill Acquisition,” Academic Emergency Medicine 15, no. 11 (2008): 1046–1057. 18785939 10.1111/j.1553-2712.2008.00218.x

[7] K. U. Wong , I. Gross , B. L. Emerson , and M. P. Goldman , “Simulated Airway Drills as a Tool to Measure and Guide Improvements in Endotracheal Intubation Preparation in the Paediatric Emergency Department,” BMJ Simulation & Technology Enhanced Learning 7, no. 6 (2021): 561–567. 10.1136/bmjstel-2020-000810 PMC8936761 35520983

[8] M. P. Goldman , A. Bhatnagar , J. Nagler , and M. A. Auerbach , “Advanced Pediatric Emergency Airway Management: A Multimodality Curriculum Addressing a Rare but Critical Procedure,” MedEdPORTAL: The Journal of Teaching and Learning Resources 16 (2020): 10962. 32908951 10.15766/mep_2374-8265.10962 PMC7473185

[9] C. C. Kennedy , E. K. Cannon , D. O. Warner , and D. A. Cook , “Advanced Airway Management Simulation Training in Medical Education: A Systematic Review and Meta‐Analysis,” Critical Care Medicine 42 (2014): 169–178. 24220691 10.1097/CCM.0b013e31829a721f

[10] C. Ng , N. Primiani , and A. Orchanian‐Cheff , “Rapid Cycle Deliberate Practice in Healthcare Simulation: A Scoping Review,” Medical Science Educator 31, no. 6 (2021): 2105–2112. 34950533 10.1007/s40670-021-01446-0 PMC8651942

[11] I. T. Gross , D. G. Abrahan , A. Kumar , et al., “Rapid Cycle Deliberate Practice (RCDP) as a Method to Improve Airway Management Skills—A Randomized Controlled Simulation Study,” Cureus 11, no. 9 (2019): e5546. 31523589 10.7759/cureus.5546 PMC6721918

[12] K. Schoppel , J. Spector , I. Okafor , et al., “Gaps in Pediatric Emergency Medicine Education of Emergency Medicine Residents: A Needs Assessment of Recent Graduates,” AEM Education and Training 7, no. 6 (2023): e10918. 38037628 10.1002/aet2.10918 PMC10685395

[13] N. Christopher , “Pediatric Emergency Medicine Education in Emergency Medicine Training Programs,” Academic Emergency Medicine 7, no. 7 (2000): 797–799. 10917331 10.1111/j.1553-2712.2000.tb02274.x

[14] K. A. Michelson , T. W. Lyons , J. D. Hudgins , et al., “Use of a National Database to Assess Pediatric Emergency Care Across United States Emergency Departments,” Academic Emergency Medicine 25, no. 12 (2018): 1355–1364. 29858524 10.1111/acem.13489

[15] J. J. Klotz , S. L. Dooley‐Hash , J. B. House , and P. B. Andreatta , “Pediatric and Neonatal Intubation Training Gap Analysis: Instruction, Assessment, and Technology,” Simulation in Healthcare 9, no. 6 (2014): 377–383. 25503532 10.1097/SIH.0000000000000057

[16] C. Legoux , R. Gerein , K. Boutis , N. Barrowman , and A. Plint , “Retention of Critical Procedural Skills After Simulation Training: A Systematic Review,” AEM Education and Training 5, no. 3 (2020): e10536. 34099989 10.1002/aet2.10536 PMC8166305

[17] J. D. Raper , C. Khoury , and A. D. Bloom , “Simulation in Emergency Medicine Graduate Medical Education: A Call to Lead,” Clinical and Experimental Emergency Medicine 10, no. 1 (2023): 107–109. 36718487 10.15441/ceem.22.413 PMC10090733

[18] T. Sawyer , M. White , P. Zaveri , et al., “Learn, See, Practice, Prove, Do, Maintain: An Evidence‐Based Pedagogical Framework for Procedural Skill Training in Medicine,” Academic Medicine 90, no. 8 (2015): 1025–1033. 25881645 10.1097/ACM.0000000000000734

[19] P. A. Thomas , D. E. Kern , M. T. Hughes , et al., Curriculum Development for Medical Education: A Six‐Step Approach (JHU Press, 2022).

[20] M. Acedo , “10 Pros and Cons of a Flipped Classroom,” Teachthought. 2013 accessed July 21, 2023, https://www.teachthought.com/learning/10‐pros‐cons‐flipped‐classroom.

[21] R. M. Ryan and E. L. Deci , “Self‐Determination Theory and the Facilitation of Intrinsic Motivation, Social Development, and Well‐Being,” American Psychologist 55 (2000): 68–78. 11392867 10.1037//0003-066x.55.1.68

