# Supplementary material for: Mastering Pediatric Airway Skills: A Procedural Simulation Using Rapid Cycle Deliberate Practice for Emergency Medicine Residents
Source: AEM Educ Train. 2026 May 11;10(3):e70180. doi: 10.1002/aet2.70180 (PMC13159709; doi:10.1002/aet2.70180)
Supplement: Supplementary file 1 — Table S1: Impact of RCDP simulation‐based educational intervention on procedural self‐competency and measured performance in pediatric airway management. Table S2: Thematic analysis of learner feedback on confidence with airway skills. [file AET2-10-e70180-s001.pdf]

Supplemental Table 1. Impact of RCDP Simulation-Based Educational Intervention on Procedural Self-Competency and Measured Performance in Pediatric Airway Management

**Procedural Self-Competency Survey**<br>*Please rate your agreement with the following statements, which best represent your level of preparedness and/or comfort level: I am comfortable with...*			
**Domain**	**Pre-Intervention** (N=39)	**Post-Intervention** (N=39)	**P-value**
Set up for infant intubations	20% Agree (8/39)<br>5% Strongly Agree (2/39)	44% Agree (17/39)<br>41% Strongly Agree (16/39)	<0.01
Perform infant intubations	18% Agree (7/39)<br>5% Strongly Agree (2/39)	41% Agree (16/39)<br>0% Strongly Agree (0/39)	<0.01
Set up for pediatric intubations	31% Agree (12/39)<br>0% Strongly Agree (0/39)	38% Agree (15/39)<br>49% Strongly Agree (19/39)	<0.01
Perform pediatric intubations	23% Agree (9/39)<br>0% Strongly Agree (0/39)	36% Agree (14/39)<br>31% Strongly Agree (12/39)	<0.01
**Observed Procedural Performance**			
**Domain**	**Pre-Intervention** (N=31)	**Post-Intervention** (N=31)	**P-value**
**Airway Equipment Set-up**<br>*"Prepare to intubate and verbalize what equipment you would like to ensure is available and functioning properly prior to performing airway intubation."*			
Select an infant face mask for BVM ventilation and connect to oxygen	68% (21/31)<br>Correctly selected	100% (31/31)<br>Correctly selected	< 0.05
Selects ETT size and checks cuff with syringe	19% (6/31)<br>Correctly selected	55% (17/31)<br>Correctly selected	< 0.05
Selects a laryngoscope (Straight blade 1) and tests the laryngoscope	65% (20/31)<br>Correctly selected	90% (28/31)<br>Correctly selected	< 0.05
Selects stylet	% (11/31)<br>Correctly selected	% (18/31)<br>Correctly selected	0.1
Prepares the $ETCO_2$ or capnography device	% (18/31)<br>Correctly selected	% (18/31)<br>Correctly selected	1
Selects OPA	13% (4/31)<br>Correctly selected	77% (24/31)<br>Correctly selected	< 0.05
Selects NPA	3% (1/31)<br>Correctly selected	39% (12/31)<br>Correctly selected	< 0.05
Articulate a backup plan for a difficult airway	35% (11/31)<br>Articulated	77% (24/31)<br>Articulated	< 0.05
**BVM & Airway Adjuncts**<br>*"Select the proper size mask and begin effective bag-mask ventilation of the infant with an appropriate rate and volume."*			
Applies BVM using E-C clamp technique with adequate chest rise (single-provider)	71% (22/31)<br>Correctly done	74% (23/31)<br>Correctly done	0.78
Able to troubleshoot with the poor chest rise	55% (17/31)<br>Correctly done	84% (26/31)<br>Correctly done	< 0.05
Inserts OPA	52% (16/31)<br>Correctly done	84% (26/31)<br>Correctly done	< 0.05
Inserts NPA	29% (9/31)<br>Correctly done	61% (19/31)<br>Correctly done	< 0.05

**Direct Laryngoscopy Procedural Steps**<br>*“Please prepare for direct laryngoscopy intubation. Next, intubate while verbalizing your technique.”*			
Proper (sniffing) positioning for pre-oxygenation	39% (12/31) Correctly done	90% (28/31) Correctly done	< 0.05
Opens the mouth with scissoring	74% (23/31) Correctly done	84% (26 /31) Correctly done	0.61
Insert the laryngoscope correctly using left hand	97% (30/31) Correctly done	97% (30/31) Correctly done	1
Retracts the tongue/soft tissue and identifies glottic structures	52% (16/31) Correctly done	94% (29/31) Correctly done	< 0.05
Inserts ETT to correct depth	42% (13/31) Correctly done	94% (29/31) Correctly done	< 0.05
Removes stylet and inflates ETT Cuff	74% (23/31) Correctly done	100% (31/31) Correctly done	< 0.05
Confirms ETT placement and performs aftercare (secures tube, selects ventilator settings, verbalizes meds)	52% (16/31) Correctly done	94% (29/31) Correctly done	< 0.05

Supplemental Table 2. Thematic Analysis of Learner Feedback on Confidence with Airway Skills

**Participant Feedback on Procedural Confidence**	
**Themes**	**Examples from Participant Comments**
Limited Clinical Exposure	*"Have not had a pediatric code thus far."*<br>*"No previous intubation experience in the clinical setting."*
High-Stakes Nature of Pediatric Airway	*"Stakes are always higher with kids. It makes everything harder."*
Value of Simulation-Based Training	*"Training through simulation definitely helps in being more comfortable."*<br>*"I enjoyed repeating the simulations to refresh my knowledge and skills."*<br>*"Airway sim was very helpful in my pediatric airway case."*<br>*"The airway sim sessions are helpful especially for those of us who have not had a lot of pediatric or infant airway emergencies."*
Need for Repetition and Skill Refreshers	*"Repetition over time was helpful."*<br>*"The more practice, the better."*<br>*"Needle crics, I have only simulated twice and don't feel comfortable."*<br>*"The RCDP and immediate feedback is practical and high yield for me"*<br>*"Sim was very helpful in my pediatric airway case last month. Was much more comfortable with the equipment and situation."*
Equipment and Setup Challenges	*"The old pediatric airway cart was very disorganized."*<br>*"Still working through how to improve the airway cart. Items slide around. This sim helps to practice getting the correct equipment."*<br>*"The airway curriculum significantly improved the process of setting up for pediatric airways."*
Cognitive Load	*"It takes more thought to decide what we need for pediatric patients (as opposed to adults)."*<br>*"This helps me go over how to appropriately prepare for a potential pediatric intubation."*
